# Robots do not get the coronavirus: The COVID-19 pandemic and the international division of labor

**DOI:** 10.1057/s41267-021-00410-9

**Published:** 2021-03-19

**Authors:** Steven Brakman, Harry Garretsen, Arjen van Witteloostuijn

**Affiliations:** 1 Faculty of Business and Economics, University of Groningen, PO Box 800, 9700AV Groningen, The Netherlands; 2 School of Business and Economics, Vrije Universiteit Amsterdam, Amsterdam, The Netherlands; 3 Antwerp Management School/Faculty of Business and Economics, University of Antwerp, Antwerp, Belgium

**Keywords:** COVID-19, risk, global value chains, labor markets

## Abstract

We assess the expected long-run consequences of the unfolding COVID-19 pandemic and use these as a platform to argue that international business (IB) as a field should expand its research agenda to study the international division of labor. The worldwide response to the COVID-19 pandemic is accelerating the shift toward the de-globalization of capital, but it will also speed up the move to a stronger globalization of labor. This paradoxical, simultaneous occurrence of de-globalization and globalization offers rich opportunities for future IB research.

## Résumé

Nous évaluons les conséquences à long terme attendues de l’actuelle pandémie de COVID-19, et les utilisons comme plate-forme pour argumenter qu’en tant que domaine, les affaires internationales (IB - *International Business*) doivent élargir leur programme de recherche afin d’étudier la division internationale du travail. La réponse à l’échelle mondiale à la pandémie de COVID-19 accélère non seulement le passage vers la dé-globalisation du capital, mais également celui vers une globalisation plus forte du travail. Cette genèse simultanée et paradoxale de la dé-globalisation et de la globalisation offre de riches opportunités pour de futures recherches en IB.

## Resumen

Evaluamos las consecuencias previstas al largo plazo de la pandemia del COVID-19 y usamos estas como una plataforma para argumentar que los negocios internacionales como un campo debería expandir su agenda de investigación a estudiar la división internacional del trabajo. La respuesta mundial de la pandemia del COVID-19 está acelerando la transición hacia la desglobalización de capital, pero también acelerará el traslado hacia una mayor globalización del trabajo. Este hecho paradójico y simultáneo de la globalización y la globalización ofrece grandes oportunidades para la investigación futura de negocios internacionales.

## Resumo

Avaliamos as consequências esperadas de longo prazo do desdobramento da pandemia COVID-19 e as usamos como plataforma para argumentar que negócios internacionais (IB) como um campo devem expandir sua agenda de pesquisa para estudar a divisão internacional do trabalho. A resposta mundial à pandemia COVID-19 está acelerando a mudança em direção à desglobalização do capital, mas também acelerará a mudança para uma globalização mais forte do trabalho. Esta ocorrência paradoxal e simultânea de desglobalização e globalização oferece ricas oportunidades para pesquisas futuras de IB.

## 概要

我们评估了正在发生的COVID-19大流行的预计长期后果, 并以此为平台论述了国际商务(IB)作为一个领域应扩大其研究议程, 以研究国际分工。全球对COVID-19大流行的反应正在加速向资本去全球化转变, 但它也将加快向更强劲的劳动力全球化转变。这种去全球化和全球化的矛盾的同时的发生为未来IB研究提供了丰富的机会。

## THE UNFOLDING LABOR MARKET CRISIS

At the time of the final drafting of this Commentary in January 2021, the COVID-19 crisis was still unfolding. Many countries that went into some form of lockdown in the spring of 2020, and stepwise liberalized their economies after the virus seemed under control, have had to reintroduce new lockdown measures because of localized new virus outbreaks. And countries that initially abstained from taking strong measures are now forced to act. Whatever the timing of the measures, the number of people infected with the new variant of the coronavirus continues to increase globally, as does the related number of deaths. Vaccines are coming, but vaccination campaigns take time to roll out. In the meantime, the virus mutates into new and more contagious variants.

On the economic front, 2020 has seen an unprecedented contraction of GDP in most countries across the world, and the outlook for 2021 is highly uncertain, at best. The IMF (2020) predicted a decrease of world real output by almost 5%, with Italy and Spain with −12.8% at the lower end and China with +1% at the higher end of the scale. However, on a monthly basis, these figures must be adjusted, and mostly downwards. In August 2020, for instance, the UK announced that the second quarter of 2020 came with a massive 20%-plus decline of the country’s gross domestic product (GDP). Growth rates are expected to recover in 2021 (a recent estimate is that world real output might grow at 5.4% in 2021), but the economic effects will be felt for a long time, and remain highly uncertain, being critically dependent upon the further evolution of the pandemic in the months and perhaps years to come. And despite this growth prediction for 2021, the level of GDP in 2021 would then still be some 6.5 percentage points lower than in the pre-COVID-19 projections of January 2020.

At the individual firm and worker level, the impact of the COVID-19 crisis has so far been very uneven. The OECD (2020) has documented the consequences of the different policy responses for workers. The impact of the COVID-19 crisis on OECD labor markets was much more severe than that of the 2008 financial crisis. In terms of the reduction in hours worked, the drop in the first 3 months of 2008 was 1.2% in OECD countries, whereas it was 12.2% in the first 3 months of the COVID-19 crisis. This is consistent with the fact that some countries went into a lockdown, implying that entire sectors have been shut down to contain the spread of the virus. This has resulted in spikes in unemployment figures in some countries, whereas reductions in hours worked were more hidden in other nations because of furloughing workers and giving income support to employees who would otherwise have been laid off. Especially vulnerable workers have been hit hard. Low-paid, flex and self-employed workers are more susceptible to job or income losses. And – as during the financial crisis of 2008 – young people face career start difficulties, as secure jobs are put on hold and internships are difficult to come by.

**Figure 1:**
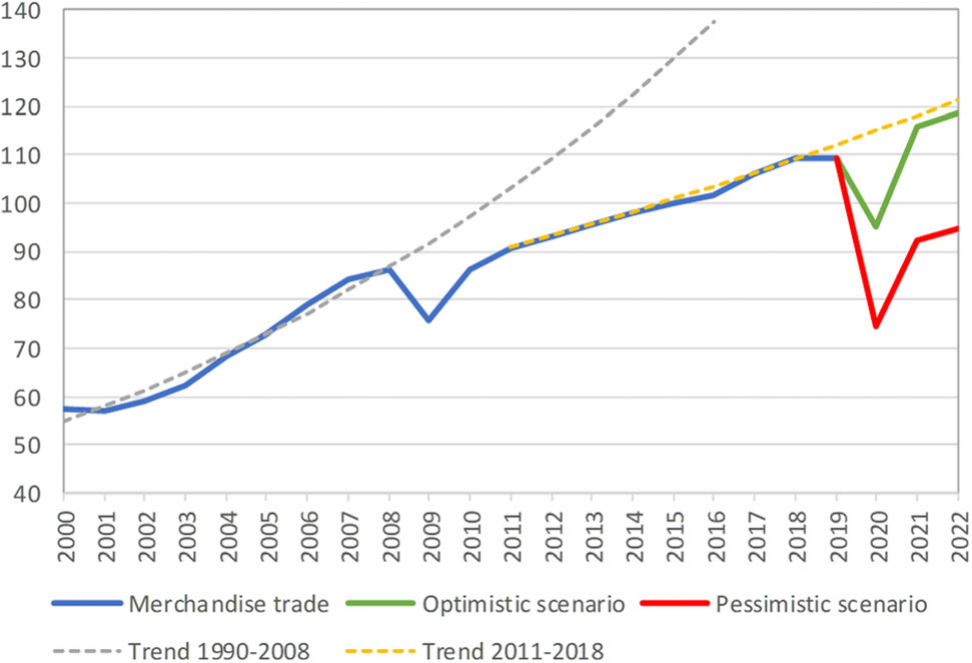
World merchandise trade volume, 2000–2022 (index, 2015 = 100). This figure is copied directly from a World Trade Organization (WTO) publication: “The WTO encourages the broadest possible dissemination of its information, particularly for educational purposes. Unrestricted official WTO documents and legal texts are free for public use.” See, for updates of trade projections, the WTO website (accessed; February, 4, 2021): https://www.wto.org/english/tratop_e/covid19_e/faqcovid19_e.htm. *Source*: WTO (2020), Press Release (https://www.wto.org/english/news_e/pres20_e/pr855_e.htm).

Whether or not the COVID-19 crisis will have a lasting negative impact on individuals and countries depends largely on the location of firms and their workers. So far, some countries have gone to considerably greater lengths than others to protect their firms and workers through government support. The firm-specific effects of the crisis also depend on the production structure of the firm, notably the degree to which firms take part in international or global value chains that were hit by disruption. With international trade and transport being disrupted because of COVID-19, many global value chains have also been affected, which has been a key driver of the sharp drop in international trade that occurred from the 2nd quarter of 2020 onwards. The contraction of international trade is thought to be more significant than the trade collapse that occurred during the Great Recession of 2009–2010 in the wake of the global financial crisis, as Figure 1 illustrates.

To add to the state of flux in which most firms and their workers across the globe now find themselves, the COVID-19 crisis has also left its mark on the internal organization of most firms, since many workers now have to work from home to a substantially larger degree than they were used to pre-corona. Estimates suggest that, in April 2020, some 62% of employed Americans worked from home. This percentage was more than double that in the pre-COVID-19 period (McKinsey, 2020). With lockdown restrictions imposed by national and regional governments, workers and management of firms had to adapt almost overnight to a new world, where working from home (WFH) is now the ‘new normal’. The extent to which the sharp uptake in WFH is here to stay remains to be seen, but, clearly, many have discovered that a surprisingly large number of organizational or even production tasks previously thought to require face-to-face interaction can also be performed in an online fashion (ILO, 2020).

Some will argue that the current COVID-19 crisis is merely a transitory situation, and that, once the dust has settled, the international economy will, just like with an ordinary recession, return to its pre-shock growth path. From this perspective, the COVID-19 crisis will ultimately not lead to structural changes in the international economy nor affect the way firms and workers conduct their activities. We strongly disagree with this perspective. In our view, the COVID-19 crisis will likely have long-lasting implications for firms and workers, in particular for those engaging in activities that are not confined to the domestic economy. Building upon the modern international business (IB) and international economics literature, we predict that, for foreign direct investment (FDI), for trade patterns, and, more generally, for the *international division of labor*, the COVID-19 crisis will turn out to be a watershed moment.

In this Commentary, we focus mainly on the international division of labor. We briefly develop a number of arguments to support the claim that the COVID-19 pandemic will lead to structural changes in the international division of labor. Our arguments are by no means the ‘final word’ on the issue, but are meant to inform fellow researchers, and also to provide some guidance to practitioners. In doing so, we will focus on two key elements. First, the COVID-19 crisis will disrupt the way international economic activities are organized across countries and firms. Second, the international “within-organization” impact of COVID-19, as an outcome of crisis practices, is that WFH will increase structurally. What does this mean in practice for the international division of labor?

First, pandemics such as COVID-19 will strengthen the ongoing trend towards automation and the robotization of work. This implies that the COVID-19 disruption will further stimulate the process of labor-saving technological change. This process will unfold globally. And to the extent that COVID-19 responses give rise to re-shoring or de-globalization, this will mainly involve re-shoring of production and not so much of jobs per se. Second, the WFH trend fueled by the COVID-19 crisis will not necessarily benefit large segments of the labor force in the OECD countries. The currently imposed WFH reveals that many service-related tasks, conventionally considered as non-tradable or non-offshore-able, can actually be performed anywhere, just like manufacturing jobs that were offshored and outsourced. This will unlock the potential for many new jobs and tasks to be performed by workers in emerging market countries, triggering a major global shift in labor demand.

Overall, the COVID-19 crisis might induce structural changes in the international economy that work against the production factor ‘labor’ as such, and particularly so for workers in developed countries. The title of our contribution, “Robots do not get the virus”, is meant to reflect this prediction. The COVID-19 crisis will make it more likely that firms across the world will accelerate the introduction of labor-saving production techniques such as robotization. This means that, in terms of the international division of ‘labor versus capital’, adaptation to crises such as the COVID-19 pandemic will favor the latter. In addition, within the context of the international division of labor, new automation techniques that make jobs and tasks less location-dependent are more likely to favor workers in emerging market economies. Our view is that the international economy will be fundamentally reshuffled, with wide-ranging consequences for countries, firms, and workers.

Below, we explore the consequences of the COVID-19 pandemic for labor as a ‘factor’ across the world. In IB, attention to labor-as-a-factor is relatively limited, as the focus is traditionally on the firm, and therefore, in classic economics’ jargon, on capital. This does not imply that the study of labor has remained absent in IB. For instance, there is a rich scholarly body of work on expatriates. However, such work is largely positioned in the realm of human resource management (HRM), rather than international labor studies. The latter focuses on analyzing the labor market as an institution. We take the current COVID-19 disruption as our platform to argue that IB would benefit from adopting the international labor market perspective, to complement the international HRM angle. Our broader claim is that IB as a field would benefit from studying labor as a ‘factor’ or ‘market’, or as an ‘institution’ or ‘system’, in addition to addressing HRM issues.

We recognize that some of the value-added of IB research results from its multidisciplinary nature. In the realm of ‘division of labor’ issues, insight from other disciplines could be included. For instance, industrial relations and labor sociology could contribute much that is of relevance from an IB perspective. In this Commentary, however, we focus only on the contribution of international labor economics.

## THE INCREASED LIABILITY OF FOREIGNNESS

Zaheer (1995) argued that firms face additional obstacles or costs when engaging in international economic activity. These costs relate to various aspects of distance between countries and their firms, not just merely physical distance and the associated transport and trade costs but also cultural and institutional distance. Distance thus broadly defined affects decision-making on whether to engage in international activity and on selecting operating modes in the case of an affirmative answer (Dunning, 1979; Dikova & van Witteloostuijn, 2007). This is very well-established in IB.

International economic activity brings potential benefits (such as increased cost efficiencies and access to new knowledge or markets), which have to be weighed against the inherent ‘liability of foreignness’. This basic trade-off is a cornerstone of both the IB and international economics (IE) literature, as it helps to determine whether a firm should actually engage in international economic activity. The contemporary IE literature, with its emphasis on the roles of firm heterogeneity, ownership, and institutional differences, in addition to the role of physical distance, has conceptually moved closer to the field of IB than was the case in the past (see Antràs, 2016, for an overview).

The concept of liability of foreignness has always been critical to the understanding of international economic activity. In fact, the ebb and flow of modern globalization since the late nineteenth century could be interpreted as a series of policy- and technology-induced changes in the liability of foreignness or, in modern IB parlance, the additional costs of doing business abroad (Hymer, 1960). Improvements in the international transport infrastructure, the lowering of tariffs, and institutional (and even cultural) convergence all led to a decrease in this liability in the first decades after the Second World War, with, as a result, a sharp increase in both FDI and trade, in tandem with the rise to prominence of the multinational enterprise (MNE). In contrast, mercantilist or protectionist policies in the 1930s or nowadays, as implemented by China, serve as a reminder that the liability of foreignness can also increase (cf. Baldwin & Evenett, 2020; Baldwin & di Mauro, 2020).

Using the terminology developed by Baldwin (2016), modern globalization since the late nineteenth century had two distinct stages. In the first stage, which lasted until the 1990s, the international division of production and labor was mainly driven by the geographical separation of production and consumption. International specialization in combination with the transport revolution of the nineteenth century made this separation possible. Baldwin (2016) calls this the first unbundling stage of globalization. Within countries, international specialization especially benefits those workers who have skills used intensively in the industries in which the country has a comparative advantage. In the resulting global division of labor, high-skilled workers in developed countries and low-skilled workers in emerging market economies were supposed to benefit from globalization.

The information and communication technology (ICT) revolution that took off in the 1990s made it possible to geographically separate the various stages in the production process itself. Various production stages became trade-able and also market-able. In line with Buckley (2009), Baldwin (2016) labels this as the stage of the second unbundling. Starting in the mid-1990s, and spurred by the participation of China in the global economy, offshoring and global value chains emerged as salient characteristics of the global economy. As with the first unbundling stage, the introduction of ICT reduced the liability of foreignness and helped firms to engage in international economic activity. On the one hand, the benefits of international specialization increased, resulting in cost reductions, the transfer of knowledge, and the opening up of new markets. On the other hand, the ‘distance gap’, both in a physical as well as in a cultural/institutional sense, was reduced. In terms of the international division of labor, this second unbundling was good news as regards wage levels and employment of high-skilled workers in developed countries and low-skilled workers in emerging market economies. However, workers in sectors that were offshore-able became more vulnerable in terms of wages and employment. For example, Autor, Dorn, and Hanson (2016) documented that employment in certain sectors of the US declined following the participation of China in the world economy.

Against this background, the first question is whether global value chains have been affected by the COVID-19 pandemic. And, related to this, has the liability of foreignness changed? If so, what are the possible implications for the international division of labor? Our answer to the first question is that the COVID-19 crisis has forced firms to re-assess the risks of their international economic activities, since the perceived liability of foreignness has increased. Producing in various international locations (across multiple continents), and being dependent on specialized value chains, may thus have increased risks because of international dependencies. The Peterson Institute for International Economics (PIIE, May 6th, 2020), for example, notes that China, as one of the main suppliers of medical equipment, has redirected Chinese-made supplies from exports to domestic usage. As a consequence, global prices for medical supplies increased substantially, as did global shortages. Experiences such as these might change future international relations. Governments are confronted with unwanted international dependencies and vulnerabilities. Hence, becoming too dependent on global supply chains might lead them to re-evaluate the net benefits of global trade and the related risks (cf. Brakman, Garretsen, & van Witteloostuijn, 2020).

However, long-term international supply chain relations may also need to be reassessed. The government-imposed closure of country borders and production plants following the outbreak of the COVID-19 pandemic has reminded MNEs that relying on globally dispersed, specialized supply chains must be associated with more realistic risk assessments than has been the case in the past. Bonadio et al. (2020) note that global supply chains can act as a transmission channel of shocks across countries, and can do so very quickly. In a study covering 64 countries and 33 sectors, they quantified some of the possible GDP consequences of COVID-19 as caused by transmission of shocks across countries via international supply chain dependencies. In a simulation with a complete global lockdown, GDP reductions would be severe, averaging 29.6%, with one-quarter of this caused by transmission through the global supply chains. However, reducing reliance on vulnerable global value chains does not make countries more resilient. Perhaps surprisingly, returning to autarky in this model does not help them to become more resilient: GDP, again, would decline by about 30.2%. The intuition here is that the pandemic is global and that re-shoring would make industries dependent on national supply chains, with national lockdowns having similar effects in absolute terms as international lockdowns.

Antràs, Redding, and Rossi-Hansberg (2020) develop an open economy model in which countries experience different phases of a pandemic. They combine the well-known gravity model from international economics that describes trade linkages with the Susceptible–Infected–Recovery model from epidemiology. They show that the transmission of pandemic-related effects is quite subtle. If countries closely resemble each other, more trade leads to more human interactions, which increases the likelihood of a pandemic. The results are such that trade can magnify the likelihood of a pandemic and the spread of the virus, the spreading of a virus being less likely in closed economies. In the opposite case, with countries that are sufficiently different from each other, more trade can reduce the likelihood of a pandemic, because the spreading risk in the more affected country is diluted in the less affected country.

Based on the above studies, we cannot determine whether a country’s resilience increases or decreases as a result of international linkages. However, it would appear reasonable to expect that firms, when deciding on the international set-up of their production process, will start to put greater weight on the reliability or resilience of these linkages vis-à-vis shocks such as COVID-19. For some, this could mean re-shoring (though not necessarily to the home country only), re-assessing just-in-time delivery, and allowing for increased slack and inventory build-up. It could also imply re-locating to countries with similar institutions and policy preferences. Whatever the precise outcome of this re-assessment, it is hard to escape the conclusion that the liability of foreignness will be affected by the COVID-19 crisis in the foreseeable future. More specifically, the liability of foreignness will, *ceteris paribus*, probably increase, which will place downward pressure on the globalization process of production, as firms (and governments) will attribute greater value on geographically closer and more reliable production processes (Brakman, Garretsen, & van Witteloostuijn, 2020). For Europe, for instance, this may imply a shift away from Asia towards Eastern Europe (Javorcik, 2020).

Note that the COVID-19 shock could be seen as merely intensifying an already existing trend. The world had become more uncertain even before COVID-19 entered the stage. In 2017, a global trade war involving the United States and China started to unfold, and weakened the WTO. In 2016, citizens in the UK decided to leave the EU by voting in favor of ‘Brexit’, which the present Conservative government is now implementing. And the European Monetary Union might face another Euro crisis in the aftermath of COVID-19, as also occurred during the financial crisis of 2008-9, with one key issue being the ability of southern European countries to repay their debts. In addition, a number of climate scientists have suggested that COVID-19 is only one example of the type of disruption that can be expected to increase in frequency and intensity in the coming decades. For instance, climate change could bring more extreme weather events, and possibly also stimulate the development of yet unknown infectious diseases. Based on the above, it could thus be argued that COVID-19 only added to the already increasing liability of foreignness. However, because the current pandemic does so very saliently, being a very visible and harsh attack with immediate effects on our way of living, this additional disruption may well push the world economy toward structural change.

Assuming that the liability of foreignness, and hence the costs of distance, will not only temporarily (in the midst of the 2020 COVID-19 crisis) but also structurally increase, what could then be the main implications for the international division of labor? At first sight, any tendency to re-shore production would appear to be good news for relatively low-skilled workers from firms in OECD countries, and would similarly be bad news for (less-skilled) workers in China and other (former) emerging market economies. The associated higher production costs in the OECD segment of the world economy would need to be balanced against the lower risks of value chain disruption.

However, there are a number of problems with the expectation that re-shoring would increase employment in OECD countries. The first problem is that a pandemic such as COVID-19 provides a strong incentive for labor-saving technological change (Seric & Winkler, 2020). Firms have an incentive to substitute capital for labor, because capital cannot be infected by the virus, at least not directly. The second problem is that this labor-saving trend was already ongoing before COVID-19 set in, in both developed and emerging market economies, in the form of the digital or industrial 4.0 revolution. Automation and robotization have been on the increase across the world (Acemoglu & Restrepo, 2018). And, unlike the technological change that occurred with the rise of the personal computer in the 1990s and 2000s, this new type of technological change is not primarily labor-augmenting (in favor of high-skilled workers), but mainly labor-saving (negatively affecting all types of workers). The third problem, which is related to the second one, is that the end of the COVID-19 crisis will not simply mean that ‘jobs will be coming back’. Firms that relied on low-cost international labor and now consider re-shoring will be incentivized to switch to more capital-intensive production to continue saving on labor costs.

However, importantly, there is also an additional, pandemic-induced change that may have long-lasting consequences for the international division of labor: The crisis response to massively stimulate working from home. We discuss this issue and its consequences for labor in the next section.

## THE WORKING-FROM-HOME SHOCK AND THE INTERNATIONAL DIVISION OF LABOR

**Figure 2:**
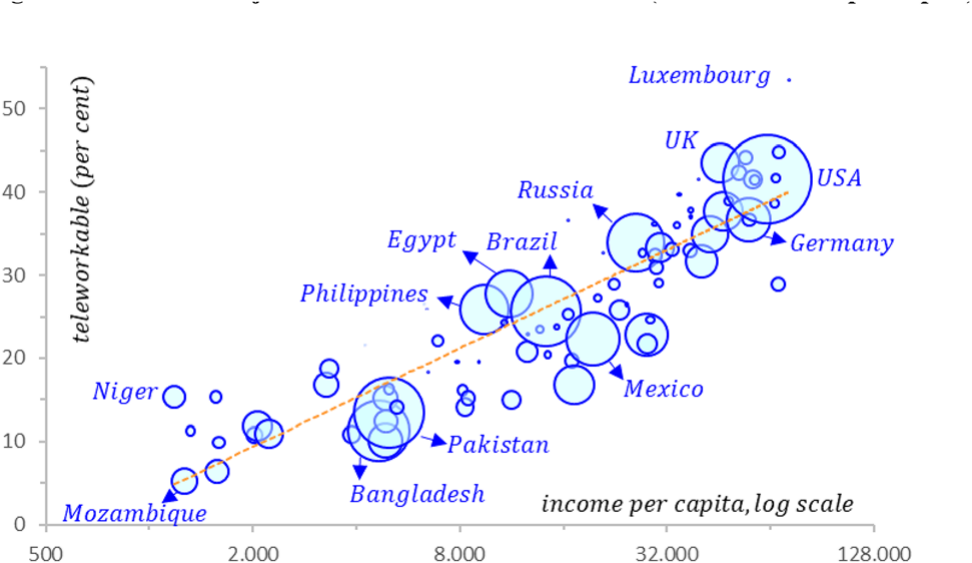
The share of jobs that can be done from home (relative to GDP per capita). Source: Dingel and Neiman (2020). We would like to thank Charles van Marrewijk for constructing this graph. The *size of the circles* is proportional to population size. The *regression line* depicts: *y* = 8.5454ln(*x*) − 55.591, with *R*^2^ = 0.7885. The countries named in Fig. 2 are selected for illustrative purposes only.

With the current COVID-19 crisis, many governments decided almost overnight that workers had to work from home whenever feasible. In countries such as the US, the UK, and Germany, and in a wide range of sectors, this actually meant that a large percentage of workers could and would start working from home. Dingel and Neiman (2020) predicted that close to 40% of all US jobs could be done from home, a percentage approximating the actual number of US workers who were forced to work from home during the spring of 2020. Typical jobs that can be done from home include, inter alia, those relating to computer and information technology occupations, education and training, and library tasks. Jobs that cannot be done from home are, for instance, those involving construction and infrastructure maintenance. Figure 2 shows the relationship between the share of jobs in individual countries that can be performed from home and income per capita.

Figure 2 reveals a positive relationship between the two variables: The more advanced an economy in terms of income per capita, the higher the share of jobs that can be done from home. This figure also suggests that developing countries and emerging markets may face greater challenges fighting COVID-19 in terms of limiting the pandemic’s negative effects on GDP.

HRM experts expect that this increase in working from home (WFH) will to a substantial degree persist after the COVID-19 pandemic wears off, structurally increasing the share of this work practice because of the perceived benefits to workers and organizations (Kniffin et al., 2021). From an organizational perspective, most research indicates that, overall, WFH has positive performance effects, and is perceived to increase productivity (Stoker, Garretsen & Lammers, 2020). This observation highlights, again, the unfortunate reality that developing countries and emerging markets may face comparatively greater structural challenges coping with the COVID-19 pandemic and its aftermath.

The ‘Zoomification’ of working relationships and tasks since the start of the COVID-19 crisis in many organizations across the globe will potentially have far-reaching implications for the international division of labor. On the one hand, one could argue that the imposed nature of WFH will make many workers and managers want to return, post-crisis, to a lower level of WFH, and to a more balanced mix with work at the office. An optimal mix (assuming this can be established) might increase productivity further and also serve the social purpose of work. On the other hand, many workers and managers may have learned that more tasks can be done remotely, away from the organization and in an effective fashion, than contemplated before the crisis. This conclusion may hold in sectors such as educational services, professional, scientific, and technical services, finance and insurance, and wholesale trade. To a lesser extent, it may also be valid for at least some tasks in industries such as manufacturing, transportation, construction, retail trade, agriculture, forestry, fishing and hunting, and food services. In the context of modularizing global value chain activities, WFH may trigger a (further) fine-slicing of tasks across industries.

Baldwin (2019) argues in his latest book, *The Globotics Upheaval,* that the rise of the ICT-driven knowledge economy will make workers engage less with tangible capital (“things”) and more with intangible capital (“thoughts”). This implies that the specific physical location for performing such work will become less important. The decoupling of the formal workplace from where work is actually performed is likely to affect the future of work (see also Haskel & Slaughter, 2018). An increasing number of tasks will be performed and coordinated without the (co-)workers involved actually being located in the same workspace or, importantly, in the same country (Baldwin, 2019).

From the perspective of the international division of labor, the effective global labor supply for any given task performed by an organization may vastly increase if workers and the factory or office can be de-coupled. This phenomenon could be called “offshoring 2.0”, in contrast to conventional “offshoring 1.0”, whereby an MNE moved to a host country to bundle firm-level knowledge with local labor. The underlying rationale for offshoring 2.0 may be identical, namely to gain access to low-cost labor, but in this instance local labor may only connect ‘virtually’ with knowledge capital in the MNE’s home country. Hence, while product markets may be subject to de-globalization, the labor market will be pushed towards higher globalization.

As to the impact of COVID-19, the unprecedented move to WFH, especially in more highly developed and service-oriented countries (see Dingel & Neiman, 2020), may provide a strong incentive for MNEs to continue to experiment with the newly discovered WFH options. The COVID-19 crisis has provided a real-world proof of concept of WFH. In many settings, apparently, workers do not have to live at commuting distance. As Baldwin (2019) argued, the labor market for these new forms of work has (or will) become global. In the IB literature. Ghemawat (2001, 2011) has long argued that most MNEs will continue to operate somewhere between the old “geography is destiny” and the “death of distance” opposites. The COVID-19 crisis, with its acceleration of the WFH trend, will be one more push in the direction of the latter option as far as labor is concerned.

## THE IB STUDY OF LABOR

The COVID-19 crisis is unique in its kind in modern history and is still unfolding, with the long-run aftermath yet to come. Inevitably, there is enormous uncertainty as to what the legacy of this crisis will be along multiple dimensions in terms of health, economic, political, organizational, and societal impacts. Despite this uncertainty, it is important to start the academic debate now on these potential impacts. Such debate will inform future research, as well as actual decision-making by firms and policymakers. And such debate will, hopefully, build upon prior research and knowledge in the quest to understand the consequences of the unfolding COVID-19 pandemic, both in the short and long run. IB research is particularly well positioned to address new opportunities for studying global and spatially differentiated impacts in this realm.

We have taken the current COVID-19 pandemic as our stepping stone to illustrate that IB could expand its research agenda regarding the ‘factor’ labor, thereby studying labor as an ‘institution’ and ‘market’ with a clear international dimension affecting the operation of MNEs. We discussed two intertwined elements, namely the COVID-19 crisis in general and the WFH dimension in particular. We have argued that the current pandemic crisis will probably have long-lasting consequences as to how MNEs organize their international activities, concluding that these consequences will not necessarily benefit workers in advanced economies.

Our main conclusion is twofold. First, pandemics such as COVID-19 will strengthen the ongoing trend toward the automation and robotization of work. This implies that COVID-19 will further stimulate the process of labor-saving technological change. Second, the WFH trend that received a unique boost from the global response to the COVID-19 pandemic will not necessarily favor labor either, at least not for large segments of the labor force in the OECD countries. In emerging market economies, labor-saving technological change will also become more feasible. This implies a paradoxical, long-term impact of the current pandemic crisis. On the one hand, the revealed vulnerability of global value chains may well strengthen the ongoing de-globalization of capital. On the other hand, the reduced importance of distance as revealed by the increasing share of WFH is likely to trigger a trend toward the globalization of labor.

This paradox is critical to future IB research. By expanding its scope to the study of the international division of labor, IB can help develop an integrated perspective on the paradoxical processes of the de-globalization of capital in tandem with the globalization of labor. What will the latter imply for the former? Will the globalization of labor further enhance the de-globalization of capital, since capital can now connect to labor in host countries without the need to move there? And how will this affect the organizational make-up, behavior, and performance of MNEs? With WFH on the rise, HRM practices may also be disrupted. WFH might also trigger the emergence of a new “global labor market” intermediary industry. In this context, we may have to return to “old” IB issues and re-examine the established answers to these classic questions in light of the disruption of the global economy induced by COVID-19. Such issues would include the role of expatriates in a globalizing labor market and the coming shape of FDI.

## Notes

1. The World Health Organization keeps track of the number of worldwide confirmed COVID-19 cases and related deaths: https://covid19.who.int/.
2. Brakman and van Marrewijk (2019) show, in the context of the great recession, that strong participation in global supply chains can also slow down a recovery after a major global economic shock.
3. This is a key reason why the EU rather than individual member states started borrowing in international financial markets in its and their fight against the economic downturn due to COVID-19.
4. See, for instance, the collection of (medical) research papers at https://www.bmj.com/communicable-diseases.
